# Immunomodulatory effects of probiotic supplementation in patients with asthma: a randomized, double-blind, placebo-controlled trial

**DOI:** 10.1186/s13223-022-00753-4

**Published:** 2023-01-02

**Authors:** Sina Sadrifar, Tannaz Abbasi-Dokht, Sarvenaz Forouzandeh, Farhad Malek, Bahman Yousefi, Amir Salek Farrokhi, Jafar Karami, Rasoul Baharlou

**Affiliations:** 1 Department of Immunology, School of Medicine, Semnan University of Medical Sciences, Semnan, Iran; 2 Cancer Research Center, Semnan University of Medical Sciences, Semnan, Iran; 3 Department of Internal medicine, Kosar Hospital, Semnan University of Medical Sciences, Semnan, Iran; 4 Department of Immunology, Pasteur Institute, Tehran, Iran; 5 Molecular and Medicine Research Center, Khomein University of Medical Sciences, Khomein, Iran

**Keywords:** Asthma, Probiotic, MicroRNA, IL-4, IFN-γ

## Abstract

**Background:**

Asthma is considered to be a chronic inflammatory disorder of the airways. Probiotics are living microorganisms that are found in the human gut and have protective effects against a wide range of diseases such as allergies. The aim of this study was to investigate the improvement of clinical asthma symptoms and changes in the expression pattern of selective microRNAs in patients with asthma and the changes in IL-4 and IFN-γ plasma levels after receiving probiotics.

**Materials and methods:**

The present study was a randomized, double-blind, placebo-controlled trial that enrolled 40 asthmatic patients. They were treated with probiotics or placebo: 1 capsule/day for 8 weeks. Pulmonary function tests, IL-4 and IFN-γ levels, and expression of microRNAs were assessed at baseline and after treatment.

**Results:**

The results showed that the expression of miR-16, miR146-a and IL-4 levels in patients with asthma after receiving probiotic supplementation was significantly reduced and miR-133b expression was increased. In addition, pulmonary function tests showed a significant improvement in Forced Expiratory Volume in 1 s and Forced Vital Capacity after receiving probiotics.

**Conclusion:**

In our study, 8-week treatment with probiotic supplementation led to reduced Th2 cells-associated IL-4 and improved Forced Expiratory Volume and Forced Vital Capacity. It appears probiotics can be used in addition to common asthma treatments.

## Introduction

Asthma is one of the most common chronic inflammatory diseases characterized by inflammation of the airways, recurrent shortness of breath, wheezing and bronchial hyperresponsiveness (BHR) eventually reversible blocking of the airway [1, 2]. The prevalence of asthma has increased in the past decades. A potential mechanism underlying this high prevalence is the microbial hypothesis which argues that less microbial exposure upregulates the cytokine production of T-helper cells type 2 (Th2), leading to an increase in allergic diseases [3]. The main available treatments currently for asthma include accurate assessment of asthma severity and the use of β2-adrenergic agonists, which are bronchodilators for acute reactions, and anti-inflammatory drugs such as inhaled corticosteroids [4].

According to the World Health Organization, probiotics are defined as “living microorganisms” that, if administered in sufficient quantities, can have beneficial and health effects on the host. The mechanisms that cause allergic diseases in early life are not yet fully understood. One possible cause is intestinal microbiota, in which the composition and structure of common bacteria interact with the developing immune system. Such interactions can affect the maturation of the immune system, which potentially leads to allergic Th2-type responses [5]. Probiotics are found in the intestines of humans. They have protective effects against a range of diseases such as allergies, tumors, diabetes, inflammation of the gastrointestinal tract and nervous system disorders. The effects of probiotics are significantly exerted by regulating cytokine gene expression, regulating the immune system, enhancing mucosal barrier function, and competing with pathogenic bacteria [6, 7].

MicroRNAs (miRNAs) are an evolutionary class of endogenous non-encoding RNAs (20–23 nt) that bind to the 3′ untranslated region of target mRNAs to modulate cell activity. MicroRNAs destroy or inhibit mRNA translation and regulate various biological processes, such as cell proliferation signal transduction, differentiation, apoptosis, regulate immune cell function, and thus maintain body homeostasis [8, 9]. Probiotics can affect host miRNA, thereby affecting many functions of the host [10]. The ability of probiotics to regulate miRNA expression is critical for maintaining gastrointestinal homeostasis. However, there are limited studies on the role of miRNAs in the regulation of intestinal microbiota as a treatment for disease. In addition, the capacity of the host to regulate intestinal microbiota is not fully understood in a miRNA-dependent manner. Given that probiotics could have beneficial effects on immune system-related miRNAs, the current study was designed to investigate the effects of probiotics on clinical symptoms, changes in cytokines and microRNAs, and pulmonary function in patients with asthma.

## Materials and methods

### Study design and subjects

The study was a double-blind parallel placebo-controlled randomized clinical trial. To calculate the sample size, we used the standard formula suggested for parallel clinical trials by considering type one error (α) of 0.05 and type two error (β) of 0.20 (power = 80%). Based on a previous study [11], we used changes in the parameters of pulmonary function test as the key variables between probiotic-treated and placebo groups. Based on this, we needed 20 participants in each group. Forty patients with asthma were enrolled with mean age of 38.62 ± 10.49 with a history of mild to moderate asthma for at least 1 year. Inclusion criteria were based on GINA (Global Initiative for Asthma) guideline [12]; a history of 2 or more episodes of wheezing within the past 6 months and/or a bronchodilator test confirming a positive response with a 12% increase in forced expiratory volume (FEV1) over 1 s [13]. Exclusion criteria included patients with lung infections such as pneumonia, coronary heart disease, lung cancer, primary and secondary immunodeficiency, other chronic diseases, patients who participated in other therapeutic studies in the past 6 months, patients who used high-dose multivitamins and probiotics within 3 months before screening, and patients who were infected with SARS-CoV-2 during the trial. Eligible patients were randomly divided into probiotic and placebo groups. Briefly, Microsoft Excel was used to create a simple random sequence. The allocation was determined using numbered, opaque and sealed envelopes. After performing the basic measurements, each participant selected an envelope. The whole randomization process was performed by a researcher who was blind and did not participate in the protocol. The patients in both groups took the same medication. Inclusion and exclusion criteria were the same for both the intervention and placebo groups. The arrival period (time of sampling) was December 2020 to June 2021. Blood samples were taken from patients before and 60 days after the intervention and used for the subsequent steps. At each visit, patients underwent a physical examination, measurement of blood pressure, heart rate, and pulmonary function, and evaluation of asthma symptoms, asthma exacerbations, and adverse events. The medications used by the patients were almost identical for both the probiotic group and the placebo group. The trial was conducted after approval by the ethics committee of Semnan University of Medical Sciences (IR.SEMUMS.REC.1398.226) and registered on the Iran Clinical Trials (http://www.irct.ir: IRCT20191220045830N1, Registered 2-8-2020). All participants gave written informed consent.

### Intervention

The intervention was started after the diagnosis of the first eligible patients. The probiotic supplements (Lactocare, Zist-Takhmir, Iran) have seven natural strains of beneficial bacteria including Lactobacillus casei 3 × 10^9^ CFU/g, Lactobacillus acidophilus 3 × 10^9^ CFU/g, Lactobacillus rhamnosus 7 × 10^9^ CFU/g, Lactobacillus bulgaricus 5 × 10^8^ CFU/g, Bifidobacterium breve 2 × 10^10^ CFU/g, Bifidobacterium longum 1 × 10^9^ CFU/g, and Streptococcus thermophilus 3 × 10^8^ CFU/g, and 38.5 mg fructo-oligosaccharide. The intervention group received one probiotic capsule per day (one capsule after lunch) and the control group consumed one placebo capsule containing 500 mg starch (Zist-Takhmir, Iran) per day at the same time for 60 days. The appearance and smell, color, shape, size, probiotic capsule and placebo were the same and were marked only with the label “A” or “B” on the box. The placebo and probiotic were packed in the same sealed boxes. The patients were instructed to keep the study medications refrigerated (between 2 and 7 °C) throughout the study. Both placebo and probiotic supplement capsule are registered by Food and Drug Administration of Iran.

### Outcome measures

The primary outcomes after treatment were asthma control test (ACT) scores, Forced Expiratory Volume in first second (FEV1), Forced Vital Capacity (FVC) and Forced Expiratory Volume in first second (FEV1)/Forced Vital Capacity (FVC) ratio. The secondary outcomes were changes in AQLQ scores (quality of life questionnaire scores), gene expression of miR-21, miR-155, miR-146a, miR-126, miR-16, and miR-133b in plasma as well as IL-4 and IFN-γ plasma levels during the 2 months’ intervention.

### RNA isolation and cDNA synthesis

Blood was collected from the intervention and placebo groups in EDTA tubes. It was centrifuged for 10 min at 500 ×g, 18 to 20 °C for plasma separation within 1 h after collection. The plasma was transferred to a fresh tube for subsequent analysis and stored at -80° C. Total RNA containing small RNA was isolated from 1 mL plasma using the BIOzol Reagent (Stem cell technology research center, Iran) according to the company protocol. After mixing plasma with BIOzol reagent, 250 µL of chloroform was added, and centrifuged at 2000 ×g at 4 ° C for 15 min. The upper aqueous phase was aspirated and mixed with 800 µL of isopropanol and incubated overnight at −20 ° C and then centrifuged at 2000 ×g at 4 ° C for 45 min. The RNA pellet was then washed with 75% ethanol, followed by centrifugation at 2000 ×g at 4 ° C for 20 min and the precipitated RNA was air-dried and dissolved in RNase-free water. The total RNA quantity and purity were analyzed Nano Drop ND-1000 spectrophotometer (Thermo Scientific, USA). cDNA synthesis was conducted by BON-RT synthesis kit (Stem cell technology research center, Iran) according to the manufacturer’s instructions. Briefly, 1 µg of total RNA, 1 uL of RT enzyme, 4 uL of buffer, 2 uL of dNTPs and nuclease-free H2O were added to yield a final volume of 20 uL. The reaction mixture was then incubated at 25 °C for 10 min, 42 °C for 60 min and at 70 °C for 10 min.

### Quantitative real-time RT-PCR

The abundance of miR-16, miR-21, miR-126, miR-133b, miR-146a, and miR-155 gene transcripts in the plasma was determined by using the Step One Plus real-time PCR system (Applied Biosystems, CA, USA) with SYBR Green PCR master mix kit (Stem cell technology research center, Iran). The relative expressions of miRNA were calculated by 2^−ΔΔct^ method using U6 as an internal reference for target miRNAs.

### Enzyme linked immunosorbent assay (ELISA)

The amounts of IL-4 and IFN-γ in the plasma of the intervention and placebo groups were measured at the same time by the same technician, using enzyme-linked immunosorbent assay (ELISA) kits (MabTagGmbH, Friesoythe, germany), according to the manufacturer’s protocol. The sensitivity of IFN-γ and IL-4 was 24 pg/mL and 2.3 pg/mL, respectively. Briefly, ELISA plates were coated with 100 µL capture antibody and incubated overnight at 4 °C. The wells were blocked with ELISA diluent for 1 h at room temperature. For cytokine detection, standards or patients’ plasma (100 µL) were added to each well, and incubated at room temperature for 2 h. Then, 100 µL of Detection antibodies was added to each well and incubated for 2 h at room temperature. After incubation, Avidin-HRP (Horseradish peroxidase) was added to each well (100 µL) and incubated for 30 min. Following this, tetramethyl benzidine (TMB) as substrate was added to each well and incubate for 1 h. The reaction was stopped with stop solution and absorption were measured at 450 nm using the microplate reader (Stat Fax 2100 Awarness, AZ, USA). Standard curves were calculated based on measurements of different concentrations of recombinant cytokines.

### Statistical analysis

All data were expressed as the mean ± SEM. Data comparison between groups and within groups was performed using paired t-test, independent t-test, Wilcoxon and Mann-Whitney tests. The correlations between different variables were tested by Spearman’s correlation analysis. Data analysis was performed with StepOne Software, Prism 8.0.2 (GraphPad v7, USA) and SPSS (SPSS, v22, USA). p-value of less than 0.05 was considered to be statistically significant.

## Results

### Baseline characteristics of participants

**Fig. 1:**
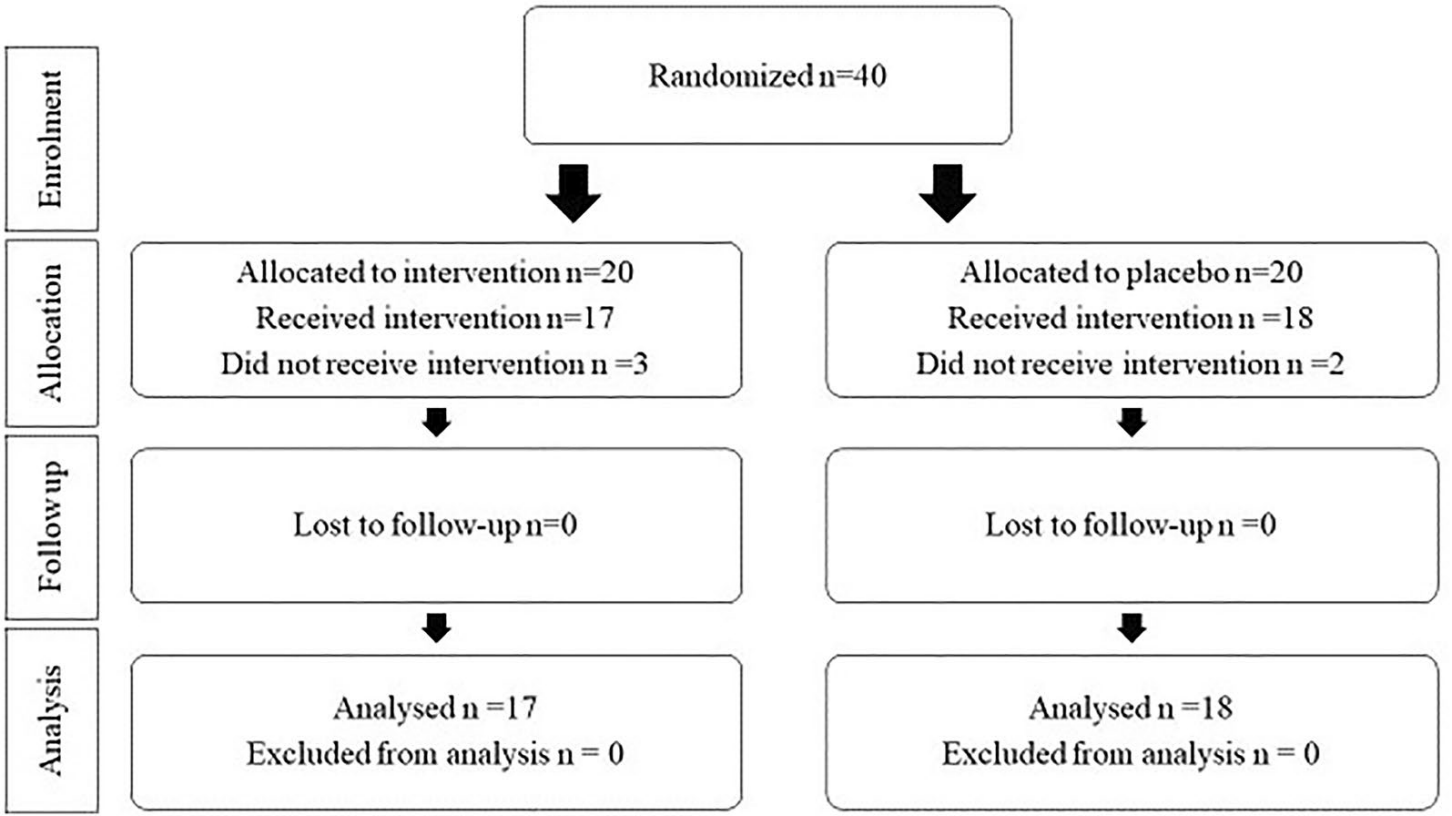
Study design

Forty patients were randomly assigned to one of the intervention or placebo groups. An accurate flow chart of study recruitment is provided (Fig. 1). In summary, five patients were excluded from the study after randomization and enrollment (three in the probiotic and two in the placebo groups). Seventeen patients in the probiotic group (f: m = 9:8) and eighteen patients in the placebo group (f: m = 5: 13) remained. There were no significant differences in patient characteristics between the placebo and intervention groups. Basic features are shown in Table 1.

**Table 1:** Baseline characteristics of the study patients

Variables	Probiotic group (n = 17)	Placebo group (n = 18)	*p*
Age	38.64 ± 9.12	38.61 ± 11.92	0.6
sex (%)	17	18	0.12
Male	8 (47.1)	13 (72.2)	
Female	9 (52.9)	5(27.8)	
Asthma family history (%)			0.40
Negative	8 (47.1)	6 (33.3)	
Positive	9 (52.9)	12 (66.7)	
Flow-up, mean (S.D.), days	80.4 ± 23.3	66.4 ± 17.9	0.14
BMI^a^, mean (S.D.), Kg/m2	25.9 ± 4.2	26.8 ± 2.7	0.10
Exposure to tobacco smoking (%)			0.35
Negative	12 (70.6)	10 (55.6)	
Positive	5 (29.4)	8 (44.4)	
Caesarean delivery (%)			0.13
Natural	10 (58.8)	6 (33.3)	
Caesarean	7 (41.2)	12 (66.7)	
Pets at home (%)			0.95
Have pet	2 (11.8)	2 (11.1)	
Not having pet	15 (88.2)	16 (88.9)	
Influenza vaccine (%)			0.92
Negative	13 (76.5)	14 (77.8)	
Positive	4 (23.5)	4 (22.2)	
Covid-19 in the intervention period (%)			0.29
Negative	17 (100)	18 (100)	
Positive	0 (0)	0 (0)	
Medications^b^			0.73
Beclomethasone + albuterol	9 (52.9)	8 (47.1)	
Fluticasone + albuterol	8 (44.4)	10 (55.6)	

^a^* BMI* Body Mass Index

^b^ Beclomethasone 250 µg two puffs q12hr + albuterol 100 µg two puffs q12hr

Fluticasone 250 µg two puffs q12hr + albuterol 100 µg two puffs q12hr

### The probiotics change pulmonary function

The spirometric measurement for pulmonary function was performed at the beginning and end of the study. The patients in the probiotic group had a significant improvement in FEV1 and FVC, after the study period compared to the baseline. In contrast, there was no significant change in the FEV1/FVC ratio in the probiotic group compared to the baseline. The results showed that the FEV1 and FVC in the probiotic group at the end of the intervention were significantly higher than at the beginning of the study (p < 0.01) and (p < 0.001), respectively (Table 2). However, FEV1 and FVC in the probiotic group were not significantly different compared to the placebo group. There were no significant changes in the level of FEV1 and FVC in the placebo group, before and after receiving the placebo. The FEV1/FVC ratio in the probiotic group did not change significantly after 60 days compared to the placebo group.

**Table 2:** Comparison of respiratory function test results in patients with asthma before and after the intervention

Variables	Probiotic group (n = 17)	Placebo group (n = 18)	Cohen’s d (95% CI)	*p*
FEV1 (L)				
Baseline	2.5 ± 0.66	2.41 ± 0.6	0.14 (−0.52 to 0.80)	0.674^t^
2nd consultation	2.7 ± 0.66	2.40 ± 0.64	0.46 (−0.22 to 1.12)	0.135^t^
Cohen’s d (95% CI)	−0.30 (−0.98 to 0.38)	0.01 (−0.65 to 0.68)		
p	0.0039^tp^	0.577^tp^		
FVC (L)				
Baseline	3.4 ± 0.69	3.2 ± 0.65	0.29 (−0.37 to 0.96)	0.449^t^
2nd consultation	3.6 ± 0.57	3.3 ± 0.68	0.47 (−0.21 to 1.14)	0.143^t^
Cohen’s d (95% CI)	−0.31 (−0.98 to 0.37)	−0.15 (−0.80 to 0.51)		
p	0.0007^tp^	0.150^tp^		
FEV_1_/FVC (%)				
Baseline	72 ± 7	73 ± 17	−0.07 (−0.74 to 0.59)	0.241^w^
2nd consultation	74 ± 11	71 ± 17	0.21 (−0.54 to 0.77)	0.285^w^
Cohen’s d (95% CI)	−0.21 (−0.89 to 0.46)	0.12 (−0.54 to 0.77)		
p	0.463^wp^	0.202^wp^		

T Student’s t and W the Wilcoxon Mann-Whitney tests for independent samples and Tp Student’s t and Wp the Wilcoxon tests for paired samples.

* FEV1* forced expiratory volume in one second,* FVC* forced vital capacity

Additionally, some subjects in the probiotic group experienced flatulence as an adverse event (data not shown).

### The probiotics alter the production of IL-4 and INF-γ levels in plasma

**Fig. 2:**
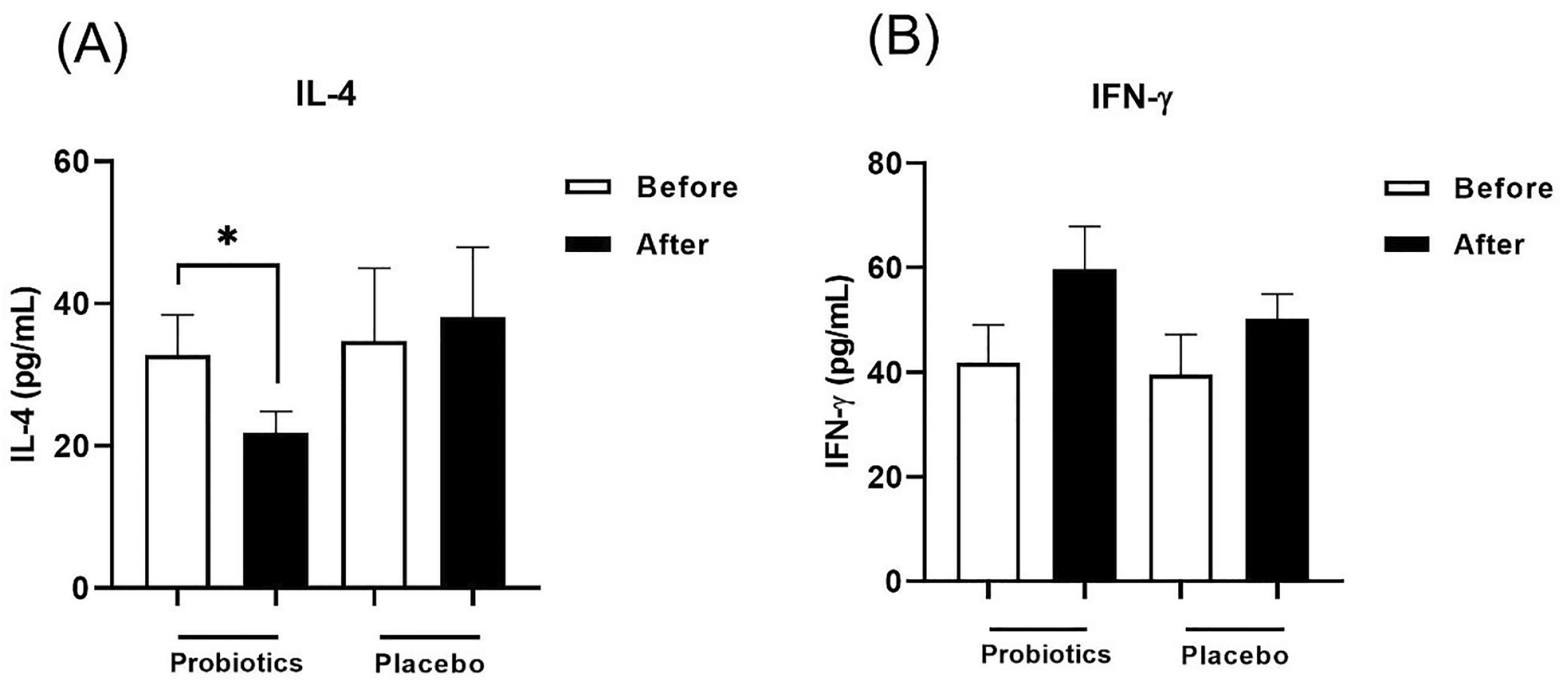
Plasma levels of IL-4 **(A)** and IFN-γ **(B)** in probiotic and placebo groups before and after intervention in patients with asthma. Bars represent the mean ± SEM. *: p<0.05

Aa shown in Fig. 2, it was observed a significant decrease in the IL-4 (Fig. 2A), following the administration of probiotic capsules for 8 weeks as compared to baseline (p < 0.05). Although IFN-γ production in the plasma was higher in the probiotic-treated group after treatment, it was not significantly different as compared to baseline (Fig. 2B). The intergroup comparison did not show any significant difference for IL-4, and IFN-γ. There was no significant change in the placebo group at the baseline and end of the intervention, neither for IFN-γ level nor IL-4.

### The probiotics alter microRNA expression profile in plasma

**Fig. 3:**
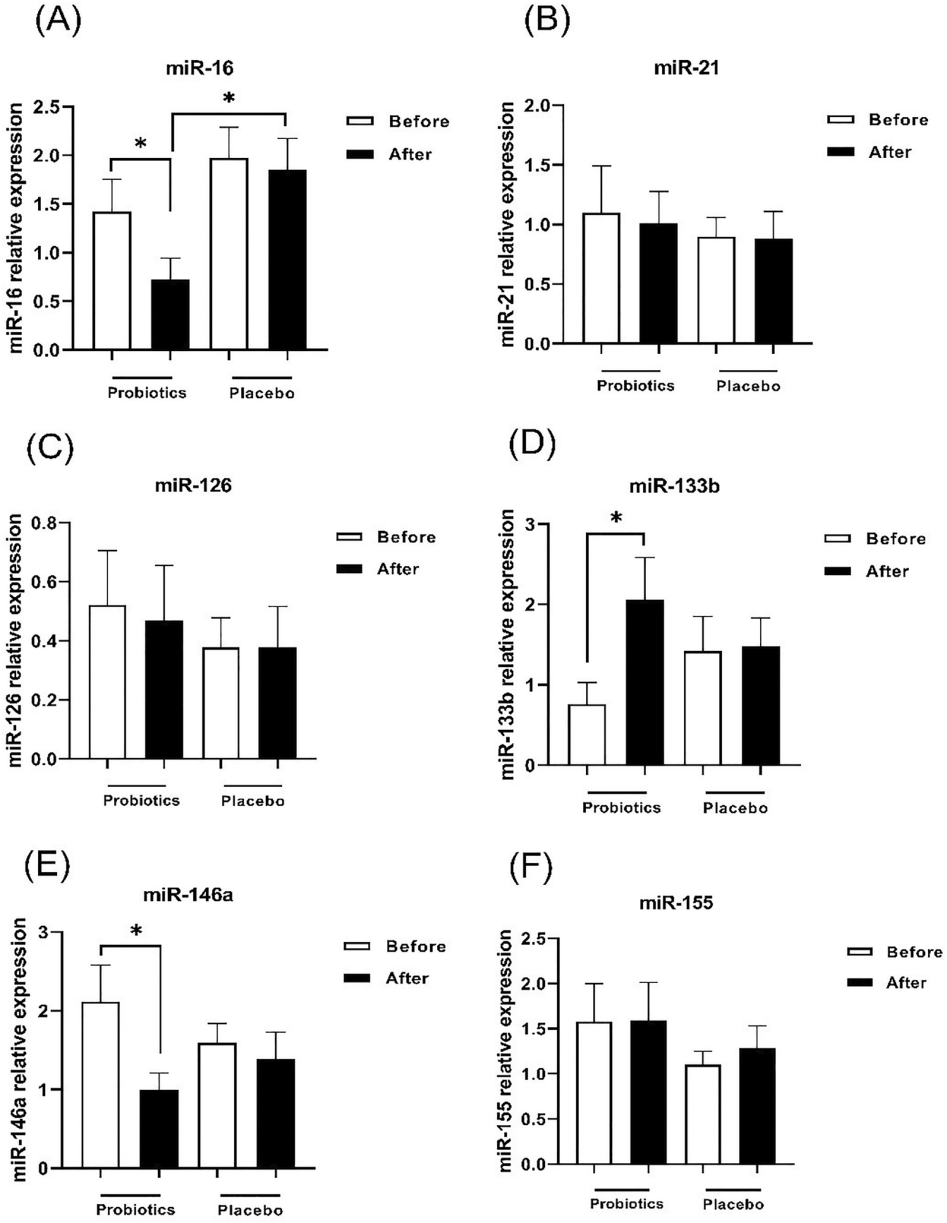
The gene expression of miRNA-16 **(A)**, miRNA-21 **(B)**, miRNA-126 **(C)**, miRNA-133b **(D)**, miRNA-146a **(E)** and miRNA-155 **(F)** in the probiotic and placebo groups before and after intervention in patients with asthma. Bars represent the mean ± SEM. *: p<0.05

In order to determine the effect of probiotic supplement on the immune system, microRNA expression in plasma was evaluated after 60 days of probiotic administration. Findings showed a significant difference in the expression of miR-146a, miR-16, miR-133b after receiving probiotics in the intervention group compared to baseline, though miR-126, miR-21, miR-155 changes were not significantly different. The patients who received probiotic supplement showed a decrease in both miR-146 (Fig. 3E) and miR-16 (Fig. 3A) as inflammation-related miRNAs in comparison with baseline (p < 0.05). Also, the expression of miR-16 in the probiotic group was significantly lower than the placebo group (p < 0.05). In addition, the expression of miR-133b in the probiotic group was increased compared to the beginning of the study (Fig. 3D) (p < 0.05).

### The probiotics improve ACT and AQLQ scores

As shown in Table 3, patients in the probiotic group had significant improvement in ACT as the asthma control test and AQLQ scores as the quality of life questionnaire scores compared to baseline. ACT and AQLQ scores in the probiotic group after receiving probiotics were significantly higher than at the beginning of the study (p < 0.001) and (p < 0.0001), respectively.

**Table 3:** Comparison of AQLQ test results in patients with asthma before and after the intervention

Variables	Probiotic group (n = 17)	Placebo group (n = 18)	Cohen’s d (95% CI)	*p*
ACT				
Baseline	15.41 ± 4.5	14.67 ± 2.3	0.06 (−0.61 to 0.72)	0.41^t^
2nd consultation	18.65 ± 4.2	16.22 ± 4.79	0.53 (−0.15 to 1.20)	0.13^t^
Cohen’s d (95% CI)	−0.73 (−1.42 to −0.03)	−0.41 (−1.08 to 0.26)		
p	0.0001^tp^	0.051^tp^		
AQLQ				
Baseline	2.4 ± 0.96	2.63 ± 0.83	−0.06 (−0.73 to 0.63)	0.50^t^
2nd consultation	3.6 ± 0.98	2.87 ± 0.80	0.79 (0.10 to 1.48)	0.01^t^
Cohen’s d (95% CI)	−1.21 (−1.94 to −0.48)	−0.29 (−0.94 to 0.37)		
*p*	0.0001^tp^	0.06^tp^		

T Student’s t and W the Wilcoxon Mann-Whitney tests for independent samples and Tp Student’s t and Wp the Wilcoxon tests for paired samples.

*FEV1* forced expiratory volume in one second,* FVC* orced vital capacity,

### Plasma miR-16 levels correlate with FEV1/ FVC

**Fig. 4:**
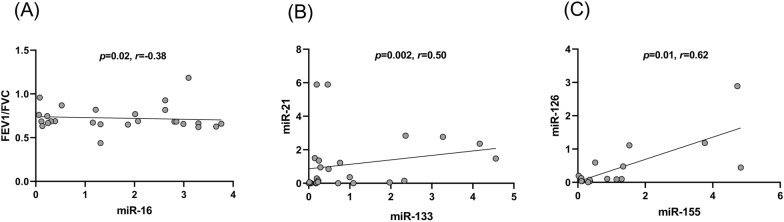
The correlation between miR-16 and FEV/FVC **(A)**; miR-133 and miR-21 **(B)**; and miR-126 and miR-155 **(C)** in patients with asthma. Data was analyzed by Spearman's rank correlation test. Scatter plot curve showed a negative correlation between miR-16 and FEV/FVC ratio (r=−0.38, p=0.02). A significant positive correlation between miR-133 and miR-21; and between miR-126 and miR-155 was shown in the plasma of patients with asthma (r=0.50, p=0.002) and (r=0.62, p=0.01), respectively

The Spearman’s correlation rank test was performed to investigate the association between miRNAs and FEV1/FVC, before and after intervention in the patients. As shown in Fig. 4, a significant negative linear correlation was found between miR-16 expression and FEV1/FVC ratio before the intervention (r=-0.38, p = 0.02) (Fig. 4 A). Also, miR-21 expression was correlated with miR-133 expression (r = 0.50, p = 0.002) before intervention (Fig. 4B) and miR-155 expression was correlated with miR-126 expression after intervention (r = 0.62, p = 0.01) (Fig. 4 C).

## Discussion

Asthma is a global health problem that can affect the quality of life [14]. There is now evidence that microbiota is associated with a variety of diseases including asthma [15]. Probiotics are living microorganisms that are present in the human gut. They have been shown to be effective in a variety of clinical conditions such as neonatal diarrhea, antibiotic-associated diarrhea, Helicobacter pylori infection, inflammatory bowel disease, cancer, autoimmune diseases and allergies [16–18]. Different strains of probiotics have been studied for their effects on asthmatic patients. In the study by Chen et al. the impact of L. gasseri A5 supplements in a short-term (2-month) trial in children with asthma aged 6 to 12 years was assessed. They found that this strain of probiotics could improve peak expiratory flow rate levels and CACT score and could significantly reduce the levels of TNF-α, IFN-γ, IL-12 and IL-13 production by peripheral blood mononuclear cells [11]. However, supplementation with L. casei [19] for 12 months or non-pathogenic Enterococcus faecalis [20] for 17 weeks had no beneficial effect on asthma. Our 8-week double-blind randomized intervention trial showed that probiotic supplementation had a significant beneficial effect on AQLQ and ACT scores, and also improved lung function (FEV1 and FVC) were seen. There is a difference in ACT and AQLQ scores in the placebo group, but the difference is not statistically significant. In this regard, it seems that the effects of probiotics on scores, especially ACT, should be examined more carefully. Measurement of the pulmonary parameters showed that there was a significant increase in FEV1 and FVC after probiotic administration in the intervention group. However, probiotic supplementation did not affect the FEV1/FVC ratio. Probiotics probably exert their effect in two different ways. In the first way, probiotics can affect the immune system with products such as metabolites, cell wall components and DNA. Probiotics can systematically affect the function of immune cells by producing SCFAs (short chain fatty acids) such as acetate, butyrate, propionate, and modulate inflammatory responses [21, 22]. SCFAs produced in the intestine enter the bloodstream and affect the immune system in remote parts of the body [23–25]. SCFAs can also affect T cell differentiation through changes in cellular metabolism and energy levels. For example, propionate and butyrate increase FoxP3 expression and inhibit HDAC (histone deacetylase) in naive T cells, causing differentiation of naive T cells into Treg CD4 + FoxP3 + cells [26–28]. In the second pathway, some cells, such as dendritic cells (DCs), are affected by probiotics. The use of probiotics in the treatment of allergic diseases is based on the ability of probiotics to modulate TLRs and identify antigens that activate DCs. This will increase the response of Th1 cells. A possible mechanism is that an antigen binds to TLRs and leads to the activation of macrophages. Active macrophages activate IL-12-inducing Th1 cells and NK cells to produce IFN-γ. This cytokine inhibits Th2 cells and prevents the formation of IgE by B cells, thereby reducing asthma symptoms. The study by Torii et al. demonstrated that the species Lactobacillus acidophilus strain L-92 could balance the immune response of Th1 and Th2 through inducing Th1-related IFN-γ, and suppressing IL-4 [29]. In accordance with previous studies, we measured IFN-γ and IL-4 level. It was shown that IL-4 levels were significantly reduced compared to the baseline. It seems that probiotics can act as Th1 adjuvants and regulate immune responses by balancing the Th1/Th2 response. Experimental and clinical evidence has shown that probiotics can regulate the host immune system or the microbial balance of the gut, leading to a reduction in allergic diseases [30].

MicroRNAs are the most prominent form of post-transcriptional gene regulation [31] and host-microbiota interaction plays a vital role in intestinal homeostasis through several mediators including miRNAs [32]. Teng et al. suggested that probiotic therapy could interfere with these interactions and affect the expression of miRNAs [33]. A study by Rodríguez et al. showed that miRNAs involved in reducing cecal inflammation were induced by the probiotic Lactobacillus plantarum Z01 [34]. Probiotics also affect the progression and pathogenesis of allergic inflammation by regulating microRNAs. Giahi et al. observed that L. rhamnosus GG inhibited the NF-κB pathway for the production of inflammatory cytokines by regulating miR-146a in DCs [35]. The probiotics directed intestinal cells to produce miRNAs with intestinal anti-inflammatory effects to act on host cells [10]. MicroRNAs, as documented in experimental and clinical studies, can be involved in the pathogenesis of asthma. Accordingly, we focused on the effect of probiotics on the modulation of miRNAs. Our study showed that treatment with multi strains of probiotic supplements reduced miR-146a and miR-16 in the plasma of asthmatic patients, and increased miR-133. However, we did not find significant changes in miR-155, miR-21 and miR-126. The miR16 inhibits the activation of the NFκB pathway by targeting IKKβ [36], thereby reduced expression of miR-16 may increase NF-κB expression and Th1 cell differentiation via regulating TCR signaling, thereby decreasing Th2- cell differentiation. Further analysis showed that miR-16 expression levels were negatively correlated with the FEV1/FVC ratio in patients before intervention. According to the results, probiotic supplement administration increased miR-133b expression, which is involved in regulating the expression of RhoA factor in bronchial smooth muscle [37]. It seems that increased miR-133 can reduce RhoA factor and leads to decreased contraction and overreaction of bronchial smooth muscle [38]. In our study patients with asthma showed a significant decrease in the expression of miR-146a after receiving probiotics. The study by Fei Li et al. showed that induction of miR-146a increased the production of IL-4, IgE and eosinophils [39].

The studies on probiotics allow us to better understand their role in the pathophysiology of organs. First, we should consider their ability to alter the expression of some microRNAs. Secondly, the effect of miRNAs on the pathogenesis of various diseases should be considered. In order to fight many diseases affecting the human body, the immune system has to be balanced. Taken together, these data suggest that live probiotics, as immunomodulatory agents, have the ability to regulate miRNA expression, which may be associated with altered Th1/Th2 balance. Pre-clinical studies have shown that modifying the microbiota could modulate the global immune response of the host, thus reducing sensitization and allergic inflammation [40]. Many studies have suggested the hypothesis that probiotics might be protective for asthma.

Our work had some limitations. Firstly, current study was conducted during the COVID-19 pandemic period, which made it more difficult to select uninfected patients. Therefore, the enrolled patients were not sufficiently large to achieve definitive conclusions and hence, further studies with large populations are warranted. Secondly, we did not take stool samples to check the differences in bacterial counts and species between the probiotic and placebo groups. Thirdly, we did not assess the count of Th1/Th2/Th17/Treg profile before and after the intervention among participants.

## Conclusion

Since in the following intervention with probiotic supplement, quality of life and pulmonary function parameters of patients were improved, and also inflammatory-associated miRNAs and Th2-related IL-4 were reduced, our findings suggested that probiotics can be used short-term for asthmatic patients but long-term consumption of probiotic supplement requires more clinical trials.

## Data Availability

All data generated or analyzed during this study are included in this article. Further enquiries can be directed to the corresponding author. The study was registered on the Iran Clinical Trials website at: (http://www.irct.ir: IRCT20191220045830N1).
